# Identification and characterization of a critical loop for the high activity of alginate lyase VaAly2 from the PL7_5 subfamily

**DOI:** 10.3389/fmicb.2023.1333597

**Published:** 2024-01-12

**Authors:** Muxuan Du, Xue Li, Weipeng Qi, Yingjie Li, Lushan Wang

**Affiliations:** ^1^State Key Laboratory of Microbial Technology, Shandong University, Qingdao, China; ^2^School of Life Sciences, Shandong University, Qingdao, China; ^3^Foshan Haitian (Gaoming) Flavoring & Food Co., Ltd., Foshan, China

**Keywords:** alginate, alginate lyase, PL7_5 subfamily, mutagenesis, molecular docking

## Abstract

As the major component in the cell wall of brown algae, alginates are degradable by alginate lyases via β-elimination. Alginate lyases can be categorized into various polysaccharide lyase (PL) families, and PL7 family alginate lyases are the largest group and can be divided into six subfamilies. However, the major difference among different PL7 subfamilies is not fully understood. In this work, a marine alginate lyase, VaAly2, from *Vibrio alginolyticus* ATCC 17749 belonging to the PL7_5 subfamily was identified and characterized. It displayed comparatively high alginolytic activities toward different alginate substrates and functions as a bifunctional lyase. Molecular docking and biochemical analysis suggested that VaAly2 not only contains a key catalyzing motif (HQY) conserved in the PL7 family but also exhibits some specific characters limited in the PL7_5 subfamily members, such as the key residues and a long loop1 structure around the active center. Our work provides insight into a loop structure around the center site which plays an important role in the activity and substrate binding of alginate lyases belonging to the PL7_5 subfamily.

## 1 Introduction

Carbohydrates are found abundantly in brown algae, accounting for 70% of the dry weight, and the remaining 30% is mainly ash (Holdt and Kraan, 2011). The cell wall of brown seaweed is composed of different types of polysaccharides, including cellulose, hemicellulose, alginic acid, fucoidan and laminarin. Notably, alginic acid, namely alginate, is the second most copious polysaccharide globally apart from cellulose and is considered a renewable resource for sustainability (Leandro et al., 2019). The two conformational isomers, β-D mannuronate (M) and α-L- guluronate (G) are randomly arranged by β-1,4-glycosidic linkages and compose alginates, leading to the formation of three types of alginate blocks, polyG, polyM, and heteropolymer polyMG (Synytsya et al., 2015). The gel formation and viscosity of the alginates are affected by the polysaccharide sizes and block types (Skjåk-Bræk et al., 2015). Different from alginates in brown algae, the polymers from bacteria are usually modified by mannuronate acetylase for the acetylation of O-2 and/or O-3 of β-D-mannuronate (Skjåk-Bræk et al., 1989; Baker et al., 2014).

The depolymerization of alginates is catalyzed by alginate lyases through β-elimination of glycosidic linkages and results in unsaturated alginate oligosaccharides (AOSs). Compared to saturated AOSs obtained by chemical methods, the unsaturated AOSs are more physiologically favorable (Liu et al., 2019; Li et al., 2020, 2021b; Xing et al., 2020). Meanwhile, enzymatic methods for the preparation of AOSs are more environmentally friendly compared to chemical and physical methods. In addition, the depolymerization of alginates by alginate lyases is more efficient due to the high enzyme activity and substrate specificity. Therefore, alginate lyases have been widely used in the complete degradation of brown algae and the production of AOSs (Cheng et al., 2020; Gao et al., 2021; Mrudulakumari Vasudevan et al., 2021; Cao et al., 2022; Lu et al., 2022; Tanaka et al., 2022; Li et al., 2023a; Takasuka et al., 2023). Moreover, the decomposition of the bacterial polysaccharide biofilm by alginate lyases is also a potential treatment for cystic fibrosis (Inoue, 2018; Dharani et al., 2020; Gao et al., 2021; Martin et al., 2021). To date, alginate lyases are classified into 12 families of polysaccharide lyases (PLs), including PL5, PL6, PL7, PL14, PL15, PL17 and PL18, and the newly identified families PL31, PL32, PL34, PL36 and PL39 (Helbert et al., 2019; Itoh et al., 2019; Liu et al., 2019). Based on cleavage specificity, alginate lyases are categorized as polyM-specific lyases (EC 4.2.2.3), polyG-specific lyases (EC 4.2.2.11), polyMG, and bifunctional lyases (EC 4.2.2.-) which are capable of degrading both polyG and polyM substrates (Osawa et al., 2005; Li et al., 2023b). According to the action mode, alginate lyases are classified into endo-type (EC 4.2.2.-) and exo-type lyases (EC 4.2.2.26). Among PL7 alginate lyases, most characterized lyases are endolytic except for two exolyase members, AlyA5 from *Zobellia galactanivorans* Dsij*^T^* (Thomas et al., 2013) and VxAly7D from *Vibrio xiamenensis* QY104 (Tang et al., 2020). Exolyases are mainly observed in oligo-alginate lyase families PL15 and PL17 (Cheng et al., 2020). In general, a number of alginate lyases are required for the full degradation of alginate (Zhang et al., 2021). However, the marine bacterium *Falsirhodobacter* sp. alg1 harbors a primary pathway for alginate decomposition, in which only single homologs of endo- and exo-type lyases are present but achieve efficient depolymerization of alginate (Mori et al., 2016). Based on three-dimensional structures, alginate lyases are divided into three types, including β-jelly roll (PL7, PL14, and PL18), (α/α)n toroid (PL5, PL15, PL17, and PL39), and β-helix fold (PL6 and PL31) (Xu et al., 2018; Li et al., 2021a). In addition to the catalytic domain, auxiliary domains are often found in alginate lyases, and carbohydrate-binding modules (CBMs) are the most common ones. They play a critical role in recognizing alginate termini (Sim et al., 2017), increasing enzyme activity and thermostability, and influencing product distribution (Li et al., 2015; Han et al., 2016; Yan et al., 2019; Yang et al., 2019; Zhang et al., 2019; Meng et al., 2021; Tang et al., 2022b). Moreover, alginate lyases harboring tandem catalytic domains are ubiquitous in many alginate-degrading bacteria and two catalytic domains exhibit distinct substrate affinities and minimal substrates (Sun et al., 2022; Wang et al., 2022).

Among the alginate lyases, PL7 alginate lyases are the largest group in nature, which can be further divided into six subfamilies, including PL7_1, PL7_2, PL7_3, PL7_4, PL7_5, and PL7_6. Except for the newly identified enzymes from PL7_6 subfamily that are observed to be only polyM-specific (Zhuang et al., 2018; Xu et al., 2020; Wang et al., 2022), enzymes from other PL7 subfamilies have broad substrate specificities, such as polyM-, polyG-, and bifunctional specificity. In addition, PL7 alginate lyases display significant differences in specific activities and product distributions although they share a similar crystal structure and a conserved active center (Cheng et al., 2020; Barzkar et al., 2022; Li et al., 2023b). However, the key factor(s) affecting the activity of PL7 alginate lyases remains unclear. In this study, a multidomain PL7 alginate lyase, VaAly2 with an N-terminal CBM32 domain and a C-terminal catalytic domain was identified from the marine microorganism *Vibrio alginolyticus* ATCC 17749. It showed a very high specific activity of about 5,000 U/mg toward sodium alginate. According to the sequence analysis, the CD domain belongs to the PL7_5 subfamily. To better understand the key amino acid residues related to the high alginate-degrading activity of VaAly2, the sequence profile of the center site, structure modeling, and molecular docking of VaAly2 and substrate were employed to reveal the distinct properties of the PL7_5 subfamily and critical factors responsible for the efficient alginolytic activity of VaAly2. Our work provides a better understanding of the PL7_5 subfamily alginate lyase.

## 2 Materials and methods

### 2.1 Strains and materials

*Escherichia coli* DH5α were acquired from Dingguo (Beijing, China) and applied as cloning hosts, whereas *E. coli* BL21 (DE3) were purchased from Tsingke (China) and exploited as expression hosts. All strains used in this work are listed in Supplementary Table 1. Lysogeny Broth (LB) medium was used to cultivate both *E. coli* strains. When needed, 50 μg/mL kanamycin was supplemented. The expression plasmid pET-28a (+) was purchased from Novagen (Darmstadt, Germany). Sodium alginate (purity: ≥ 98%) was obtained from Sigma (USA). PolyM (purity: ≥ 97%) and PolyG (purity: ≥ 97%) with the degree of polymerization ranging from 27 to 37 were obtained from BZ Oligo Biotech Co., Ltd. (Qingdao, China). All chemicals and reagents utilized in our research were of the highest grade.

### 2.2 Bioinformatics analysis

The gene coding for VaAly2 (GenBank accession number of *VaAly2* gene: N646_4462, protein accession number: AGV20271.1) was identified in the genome of *V. alginolyticus* ATCC 17749 (GenBank accession numbers: CP006718 and CP006719). The conserved domains of VaAly2 were calculated by SMART^1^ (Letunic et al., 2021). The residues coding for a putative signal peptide were analyzed by the SignalP 6.0 server^2^ (Teufel et al., 2022). The theoretical molecular weight (MW) was calculated by the ExPASy server of the Swiss Institute of Bioinformatics.^3^ The amino sequence alignment was performed using ClustalW^4^ (Larkin et al., 2007) and further visualized by ESPript 3.0^5^ (Robert and Gouet, 2014). The phylogenetic tree was established according to the sequence alignment on MEGA X (Auckland, New Zealand) (Kumar et al., 2018) through the neighbor-joining method, and 1,000 times of bootstrap analysis was conducted. The reference alginate lyases were obtained from the CAZy database^6^ (Drula et al., 2022). The protein homology modeling was performed using SWISS-MODEL^7^ (Waterhouse et al., 2018).

AlphaFold2 (London, UK) was used to predict the structure of VaAly2 (Jumper et al., 2021), and the open source code of this software is available from GitHub.^8^ The running conditions and hardware environment of AlphaFold2 were built by our laboratory. As the software requires high computing power, the High Performance Computing Cloud Platform of Shandong University^9^ was used for protein structure computing. After running, the highest scoring conformation was selected for subsequent analysis based on the evaluation pLDDT ranking. Finally, structures were visualized and analyzed with PyMOL Version 2.1.1.

Residues within 5 Å around the active site were selected as the composition of the active site architecture (Wu et al., 2018; Zhang et al., 2021) and sequence profile of the active site architecture of the whole PL7 family was developed on WebLogo^10^ (Crooks et al., 2004) with AlyB (PDB:7W12) designated as the template.

### 2.3 Site-directed mutagenesis

The signal peptide-free VaAly2 gene was amplified and ligated into *Eco*RI/*Xho*I-digested pET-28a (+) to generate plasmid pDMX01. Site-directed mutagenesis was conducted following a PCR-based method (Weiner et al., 1994). PCR conditions were operated as previously reported in our laboratory (Wu et al., 2018, 2020). The primers used for gene cloning and mutagenesis can be found in Supplementary Table 2. After digestion with *Dpn*I (Thermo Fisher Scientific, Waltham, MA, USA), the purified PCR fragment was transformed into the competent *E. coli* DH5α cells. Plasmid was extracted with the TIANprep Plasmid Kit (Tiangen Biotech Co., Ltd., Beijing, China) and sequenced (Tsingke, Qingdao, China) to validate the mutations. All plasmids constructed are available in Supplementary Table 1.

### 2.4 Protein expression and purification

After transformation of the recombinant plasmids into *E. coli* BL21(DE3), the cells were cultivated in LB medium with addition of 50 μg/mL kanamycin at 37°C and 200 rpm before the optical density at 600 nm reached 0.6–0.8. After 20 h of cultivation at 16°C by induction at 0.1 mM of isopropyl-β-D-thiogalactopyranoside (IPTG), the cells were centrifugated and the pellets were resuspended in the lysis buffer [50 mM Tris–HCl, 300 mM NaCl (pH 9.0)] and were further disrupted by an ultrasonic homogenizer. After centrifugation at 10,000 × *g* for 10 min, the crude enzyme solution was purified by loading onto a Ni-NTA Sepharose column (GE Healthcare, USA). The eluent was desalted with a PD-10 column to eliminate imidazole. Sodium dodecyl sulfate-polyacrylamide gel electrophoresis (SDS-PAGE) was conducted to examine the purified enzymes, and a NanoPhotometer N60 (Implen, Germany) was used to quantify the enzyme concentrations. Furthermore, the oligomeric state of VaAly2 was examined by the size-exclusion chromatography using a HiLoad 16/600 Superdex 200 prep-grade column (GE Healthcare, USA) in 50 mM Tris–HCl (400 mM NaCl, pH 8.0) with a flow rate of 1 mL/min. Myoglobulin (17 kDa), ovalbumin (44 kDa), human albumin (66 kDa) and IgG (158 kDa) from GE Healthcare were used as protein size standards.

### 2.5 Enzyme activity and biochemical characterization

The activities of VaAly2 toward different substrates and those of mutants toward sodium alginate were determined based on the ultraviolet absorption spectrometry method (Wong et al., 2000). In brief, a mixture containing 180 μL of 3 mg/mL substrate solution [50 mM Tris–HCl, 300 mM NaCl (pH 9.0)] and 20 μL of enzyme was incubated at 30°C for 10 min. After termination by boiling and ice-cooling for 10 min, respectively, the alteration in the absorbance at 235 nm was monitored. One unit (U) was specified as the quantity of enzyme causing an increase by 0.1/min in A_235_.

The optimum temperature was obtained by measuring the activity at multiple temperatures (10, 20, 30, 40, 50, 60°C) at pH 9.0. Various buffers [50 mM phosphate-citrate (pH 3.0–6.4), 50 mM Tris–HCl (6.8–9.0) and 50 mM glycine-NaOH (pH 9.5–12.0)] were used for the determination of the optimum pH at 30°C. The effect of NaCl on enzyme activity was investigated by quantifying the activity at a series of NaCl concentrations ranging from 0 to 1.0 M (0, 0.05, 0.1, 0.2, 0.3, 0.4, 0.5, 0.6, 0.7, 0.8, 1.0 M) at pH 9.0 and 30°C. To examine the effects of metal ions and other chemical agents, enzyme activities were determined in substrate solution with addition of various chemical compounds (K^+^, NH_4_^+^, Ca^2+^, Co^2+^, Cu^2+^, Fe^2+^, Mg^2+^, Mn^2+^, Ni^2+^, Sn^2+^, Zn^2+^, Fe^3+^, EDTA) to a final concentration of 1 mM.

### 2.6 Degradation product analyses

A mixture (200 μL) with 0.043 μg of VaAly2 and 0.6 mg sodium alginate was incubated in the buffer [50 mM Tris–HCl, 300 mM NaCl (pH 9.0)] at 30°C for 24 h. After termination by boiling for 10 min and centrifugation at 10,000 × *g* for 10 min, 100 μL unpurified oligosaccharide products degraded by VaAly2 were collected at different reaction time points.

To analyze the degradation pattern during the whole reaction, the oligosaccharide solutions were assayed by a gel filtration column. First, the collected product mixture was passed through 0.22-mm filters and centrifuged at 10,000 × *g* for 15 min. Then, the final products were tested with a Superdex peptide 10/300 GL gel filtration column (GE Healthcare, Madison, WI, USA) equilibrated with 0.2 M NH_4_HCO_3_ and were further subjected to fast protein liquid chromatography (FPLC) analysis at 235 nm. Meanwhile, the oligosaccharide mixture was also examined by electrospray ionization mass spectrometry (ESI-MS) set in the negative ion mode with a scanning mass from 0 to 2,000 m/z.

### 2.7 Enzyme kinetics

The kinetic parameters of VaAly2 and its mutants were determined under standard conditions using 20 of μL enzyme and 180 of μL sodium alginate at various concentrations (0.5, 0.7, 0.9, 1.2, 1.5, 1.8, 2.2, 2.6, 3.0, 6.0, 10.0 mg/ml) for 5 min. The concentrations of the products were quantified by recording the increase in A_235_ and the molar extinction coefficient of 6150 M^–1^ cm^–1^ was used for the reaction products (unsaturated uronic acids) (Farrell and Tipton, 2012; Swift et al., 2014). Velocity (*V*) at different substrate concentrations was determined as previously reported (Zhu et al., 2017). The alginate molar concentrations and their corresponding velocities were processed on GraphPad Prism (version 9.3.1 for Mac OS, GraphPad Software, Boston, Massachusetts USA, www.graphpad.com) for the determination of the kinetic parameters *K*_*m*_ and *V*_max_ by fitting the data non-linearly into the Michaelis-Menten equation. The ratio of *V*_max_ to enzyme concentrations (E) was calculated to determine the turnover number (*k*_cat_) (Zhu et al., 2019).

### 2.8 Circular dichroism spectra

Circular dichroism spectra was used to detect the secondary structural changes of VaAly2 and its mutants (Whitmore and Wallace, 2008). The concentrations of VaAly2 and its mutants were adjusted to 0.25 mg/mL in 200 μL of phosphate-buffered saline (PBS), and the temperature parameter, scanning rate and path length of a J-1500 CD Spectrophotometer (JASCO, Tokyo, Japan) were set to 25°C, 200 nm/min and 0.1 cm, respectively. Circular dichroism spectra were collected at the wavelength from 200 to 250 nm.

### 2.9 Molecular docking

Docking of a nonameric oligosaccharide onto VaAly2 was performed on Autodock Vina (Trott and Olson, 2010). Hydrogen bonds and charges were added to the ligand and receptor, and the grid box was carefully assigned before the program was operated. Further knowledge of the molecular interactions was achieved through visualization of the results on PyMOL Version 2.1.1 where enzyme structures and interactions between key residues and substrate can be thoroughly examined.

## 3 Results

### 3.1 Sequence analysis of VaAly2

**FIGURE 1:**
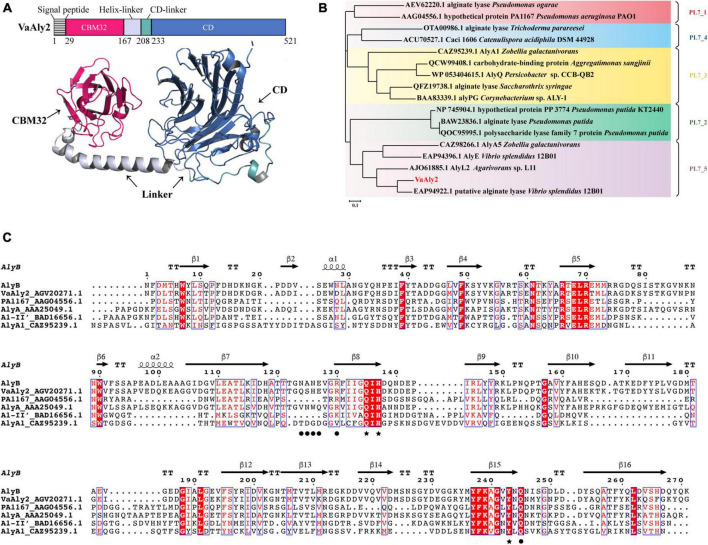
Sequence analysis of the alginate lyase VaAly2 from *Vibrio alginolyticus* ATCC 17749. **(A)** Modular analysis and protein structure prediction with AlphaFold2. CD, catalytic domain; CBM, carbohydrate binding module. **(B)** Phylogenetic analysis of VaAly2. The phylogenetic tree was established according to the sequence alignment on MEGA X through the neighbor-joining method, bootstrap analysis was conducted by 1,000 repetitions. **(C)** Multiple sequence alignment of VaAly2 with other characterized PL7 alginate lyases. Black stars indicate the amino acid residues at the catalytic center site. Black circles indicate the amino acid residues selected for mutagenesis.

In the genome of *V. alginolyticus* ATCC 17749, five genes coding for alginate lyases were identified, including 3 PL7 alginate lyases (VaAly1, VaAly2, VaAly3) and 2 PL17 oligoalginate lyases (VaAly4, VaAly5). Our preliminary experiment revealed that VaAly2 had the highest alginolytic activities toward different alginate substrates among the five lyases. Therefore, the function of VaAly2 was further studied to explore its high degradation mechanism of alginate. According to the modeled structure, VaAly2 is a multidomain alginate lyase and contains a predicted 28-residue signal peptide (SP, Met^1^-Asp^28^), an N-terminal CBM32 domain (Pro^29^-Val^166^), a C-terminal PL7 domain (CD, Met^233^-Gly^521^) and a linker between CBM32 and CD domains (Asn^167^-Ala^232^) (Figure 1A). Phylogenetic analysis revealed that VaAly2 CD belongs to the PL7_5 subfamily (Figure 1B). Sequence alignment revealed that VaAly2 contains three typical PL7 conserved domains (R*E*R, Q*H, and YFKAG*Y*Q) that form its active center for substrate binding and catalysis (Figure 1C), which was firstly demonstrated in the alginate lyase ALY-1 (Osawa et al., 2005) and A1-II’ (Yamasaki et al., 2005). The protein homology modeling result showed that VaAly2 shares the highest identity of 71.94% with the crystal structure of alginate lyase AlyB from *Vibrio splendidus* OU02 (Lyu et al., 2018), which functions as a monomer.

**FIGURE 2:**
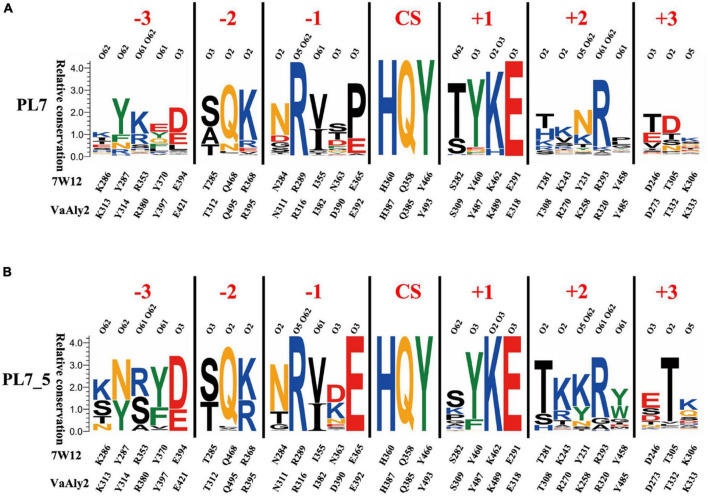
Sequence profiles of PL7 family **(A)** and PL7_5 subfamily **(B)**. The sequence profiles of panels **(A,B)** were obtained by using all candidate PL7 and PL7_5 enzymes in the CAZy database, respectively. In each sequence profile, the ordinate indicates the relative conservation degree, and the abscissa indicates the PDB ID of the template structure and the type and sequence number of each amino acid residue. Each type of amino acid is shown by abbreviated letters with a corresponding color (KRH, blue; DE, red; NQ, orange; WFY, green; others, black), and the same color suggests similar physicochemical properties. The locations of ligand atoms interacting with the residues at each subsite are shown at the bottom. CS indicates the cleavage site. For each protein structure, residues within 5 Å around the active site are shown as the composition of the active site architecture (Wu et al., 2018; Zhang et al., 2021).

To further determine the characteristics of the PL7_5 subfamily, sequence profiles of PL7 and PL7_5 were obtained by using the respective enzymes available in the CAZy database. As shown in Figures 2A, B, the VaAly2 CD contains the highly conserved amino acid residues HQY in the PL7 family to form the active center site for substrate catalysis (Yamasaki et al., 2005). In addition, some conserved amino acid residues, including Arg^316^ and Glu^318^ were also observed, which are likely involved in substrate binding. Notably, one amino acid residue Glu^392^ in the active center is fully conserved in PL7_5 (Figure 2B), and alternatively, proline is more commonly observed in the whole PL7 family (Figure 2A).

### 3.2 Heterologous expression and purification of VaAly2

**FIGURE 3:**
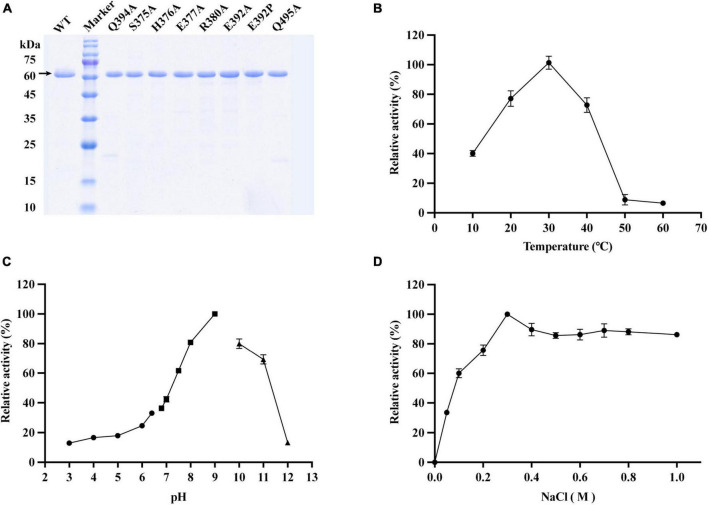
Effects of different enzymatic reaction conditions on the activity of VaAly2 toward sodium alginate. **(A)** SDS-PAGE analysis of active, purified VaAly2 and its mutants. VaAly2 protein with the molecular weight of about 62 kDa is indicated by an arrow. **(B)** The optimal catalytic temperature of VaAly2. **(C)** The optimal catalytic pH of VaAly2. Buffers prepared by 50 mM phosphate-citrate (pH 3.0–6.4) are indicated by circles, buffers prepared by 50 mM Tris–HCl (6.8–9.0) are indicated by squares, and buffers prepared by 50 mM glycine-NaOH (pH 9.5–12.0) are indicated by triangles. **(D)** The optimal NaCl concentration for VaAly2 activity. The results from representative experiments were examined in triplicate. Values are given as the means ± standard deviations.

According to the modeled structure and the sequence alignment, VaAly2 without its signal peptide was constructed and expressed in *E. coli* BL21 (DE3). The recombinant VaAly2 was purified by NTA-Ni Sepharose affinity chromatography and migrated as a single band of presumably 62 kDa on the SDS-PAGE (Figure 3A). The total VaAly2 protein yield from 1 L LB culture was approximately 10.7 mg. The size-exclusion chromatography on a HiLoad 16/600 Superdex 200 prep grade column (GE Healthcare, USA) was used to examine the oligomeric state of VaAly2. As shown in Supplementary Figure 1, VaAly2 eluted as a single peak with the molecular weight of ∼60 kDa, indicating it functions as a monomer in solution.

### 3.3 Biochemical properties of VaAly2

#### 3.3.1 Effect of temperature and pH

As shown in Figure 3B, the optimum activity of VaAly2 was observed at 30°C, and when the temperature was at 10°C, only 40% of activity was retained. The alginolytic activity of VaAly2 was hardly observed when the temperature was higher than 50°C. When assayed at different pH values, VaAly2 exhibited the maximum activity at pH 9.0 (Figure 3C). The activity was significantly decreased with reduced pH values, and VaAly2 retained only 10% of activity at pH 3.0. Under alkaline conditions, VaAly2 retained 80% of activity at pH 8.0 and pH 10.0 while only 10% of activity was retained at pH 12.0. Taken together, our data indicated that VaAly2 is a medium temperature and alkaline alginate lyase.

#### 3.3.2 Effect of NaCl

Since a number of alginate lyases from marine microorganisms showed a Na^+^-dependent activity (Cheng et al., 2020), the activity of VaAly2 was measured at various NaCl concentrations to verify the optimum condition. As shown in Figure 3D, no activity was observed when NaCl was absent, suggesting that VaAly2 is NaCl-activated. The activity of VaAly2 reached a plateau when the concentration of NaCl was higher than 300 mM, implying that VaAly2 is halophilic (Figure 3D).

#### 3.3.3 Effects of other chemical compounds

Apart from NaCl, the effects of other chemical compounds on VaAly2 were also examined. As shown in Table 1, K^+^, NH_4_^+^, and Ca^2+^ alone increased the activity of VaAly2, Mg^2+^ had no obvious effect on the activity, Fe^2+^ and Sn^2+^ slightly decreased the activity, Mn^2+^ Co^2+^, Ni^2+^, and Cu^2+^ significantly reduced the activity, and Zn^2+^ and Fe^3+^ completely inhibited the activity of VaAly2. Unexpectedly, VaAly2 retained more than 20% activity in the presence of 1 mM EDTA. Overall, Na^+^, K^+^, and Ca^2+^ have significant influences on the catalytic efficiency probably resulting from the special marine environment, and a hypothesis has been raised in other marine-derived alginate lyases (Zhu et al., 2019; Li et al., 2020; Yang et al., 2020; Meng et al., 2021; Long et al., 2022). Other high-valence cations might interact with residues located in the active center and disrupt the enzyme-substrate binding, therefore leading to distinct inhibition of enzyme activity.

**TABLE 1:** Effect of chemical compounds on the activity of VaAly2.

Chemical compounds	Relative activity (% ± S.D.)	Chemical compounds	Relative activity (% ± S.D.)
CK	100.00 ± 1.07	ZnSO_4_	2.56 ± 1.22
KCl	121.31 ± 2.80	FeSO_4_	78.29 ± 1.13
NH_4_Cl	125.56 ± 1.28	NiSO_4_	39.39 ± 0.88
MgSO_4_	104.54 ± 0.82	CuSO_4_	25.07 ± 1.33
CaCl_2_	117.68 ± 1.49	SnSO_4_	92.70 ± 1.37
MnCl_2_	38.09 ± 1.04	FeCl_3_	0
CoCl_2_	42.31 ± 1.70	EDTA	25.43 ± 1.86

#### 3.3.4 Substrate specificity

**FIGURE 4:**
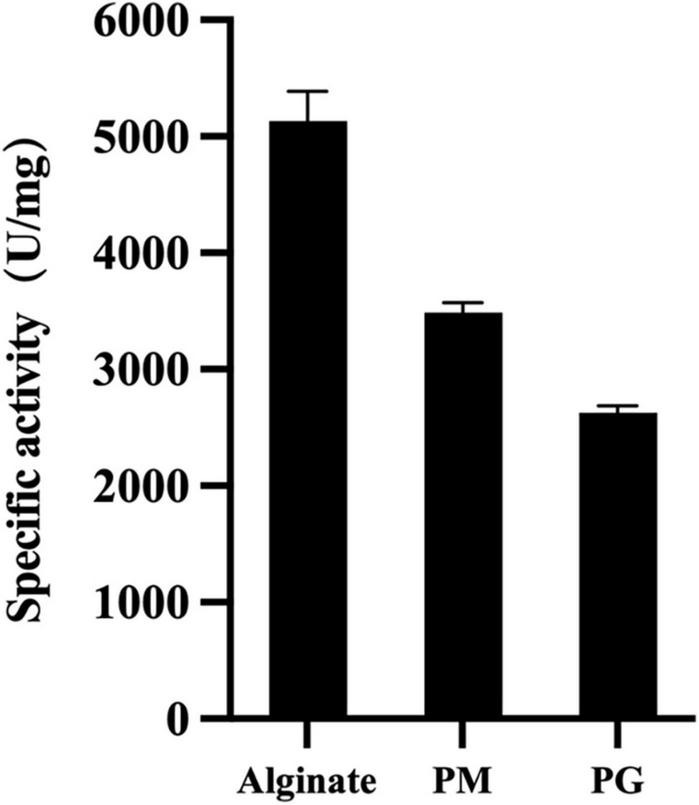
Substrate specificity of VaAly2 toward sodium alginate, polyM, and polyG, respectively. The results are shown from triplicate experiments and are given as the means ± standard deviations.

To determine VaAly2 substrate specificity, the activities toward different alginate substrates were measured. As shown in Figure 4, VaAly2 displayed the highest activity toward sodium alginate, with a specific activity of 5,133 ± 117 U/mg followed by polyM with a specific activity of 3,488 ± 87.1 U/mg and the lowest activity was observed toward polyG with a specific activity of 2,630 ± 56.9 U/mg, indicating that VaAly2 is a bifunctional lyase (Xu et al., 2018).

#### 3.3.5 Degradation products

**FIGURE 5:**
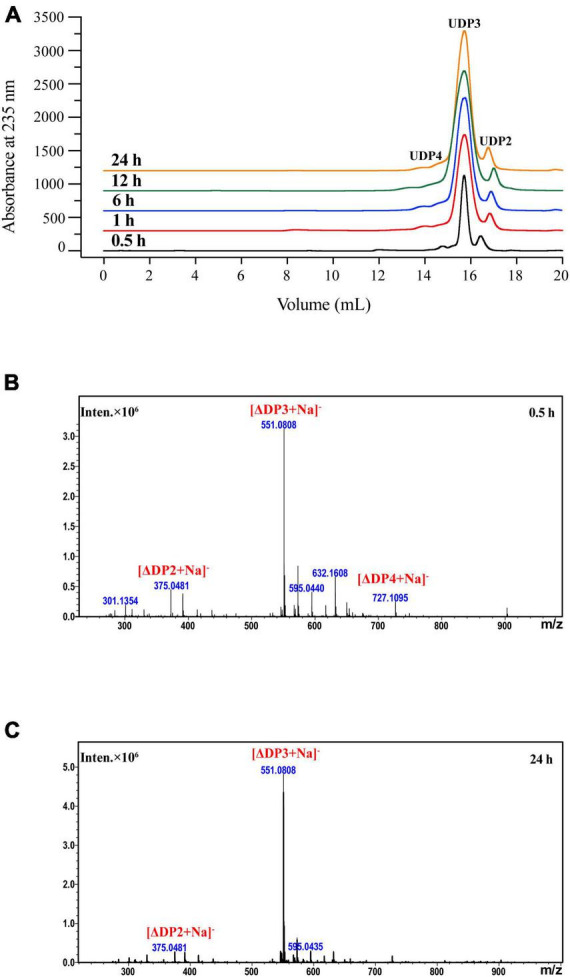
Analysis of the degradation products of VaAly2 at different reaction times. **(A)** FPLC analysis. **(B,C)** ESI-MS analysis.

The products produced by VaAly2 after different catalytic times were analyzed by FPLC and ESI-MS (Wang et al., 2022). As shown in Figure 5A, trisaccharide contributed to the major proportion of the degradation products at all tested time points. After 0.5 h of reaction, three peaks were observed at 14.78, 15.71 and 16.43 mL representing DP2, DP3, and DP4, respectively (Figure 5B). When VaAly2 interacted with sodium alginate for 24 h, only products DP2 and DP3 were detected by using ESI-MS (Figure 5C). Taken together, these data revealed that VaAly2 functions by an endolytic action mode and produces various oligosaccharides as the final products with trimers being the main one.

### 3.4 Molecular docking analysis of VaAly2

**FIGURE 6:**
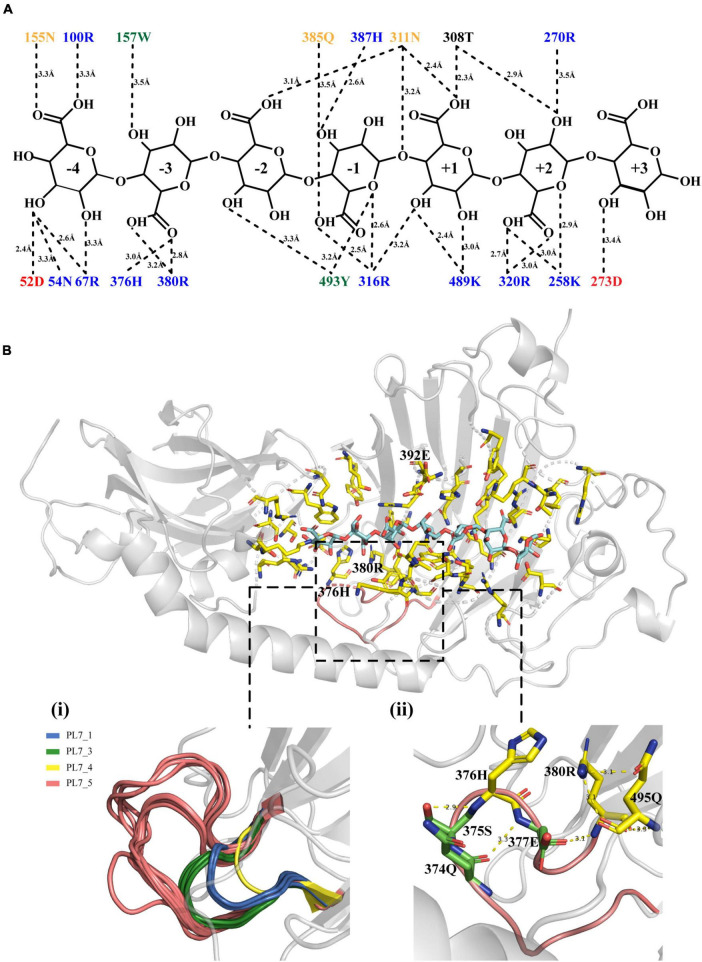
Molecular docking results. **(A)** The interaction between VaAly2 and polyG substrate. Each amino acid is shown with a corresponding color (KRH, blue; DE, red; NQ, orange; WFY, green; others, black) indicating similar physicochemical properties, and the dashed lines represent polar interactions along with the distance information between the amino acid residues and the ligand. **(B)** Structure alignment of loop1 among alginate lyases belonging to different PL7 subfamilies and detailed structure of loop1 from VaAly2. The G9 substrate is shown in blue sticks while residues within 5 Å around the substrate are presented in yellow sticks. Although the reported G9 was used for molecular docking, 7 sugar units can occupy the whole catalytic groove, which has been observed in AlyB from *Vibrio splendidus* OU02 (Lyu et al., 2018). Therefore, only 7 sugar units are shown in the figure.

To better understand the key amino acid residues responsible for the high alginate-degrading activity of VaAly2, molecular docking analysis was performed using AutoDock Vina. A nonameric polyG (G9: guluronate nonasaccharide) obtained from PDB (ID: 7W12) showed the best interaction with the whole active-site center of the VaAly2 protein, whereas the other known ligands are too small to fit into the whole catalytic groove. The docked complex was finally selected by the criteria of interacting energy combined with the geometrical matching quality. The results in Figure 6 indicated that the alginate oligosaccharide is capable of interacting with the two domains (CBM32 and catalytic domain), in accordance with the observation in Alg7A (Zhu et al., 2019), Aly01 (Meng et al., 2021) and AlyB (Zhang et al., 2022). The amino acid residues probably interacting with alginate are Lys^258^, Arg^270^, Asp^273^, Thr^308^, Asn^311^, Arg^316^, Arg^320^, His^376^, Arg^380^, Gln^385^, His^387^, Lys^489^, and Tyr^493^ positioned in the groove of CD, and Asp^52^, Asn^54^, Arg^67^, Arg^100^ Asn^155^, and Trp^157^ positioned on the edge of CBM32 adjacent to CD (Figures 6A, B). They could form hydrogen bonds with the substrate with distances between 2.3 and 3.5 Å. Structural alignment of alginate lyases from different PL7 subfamilies showed that one loop named loop1 located around the active center is varied among different subfamilies, and enzymes belonging to the PL7_5 subfamily contain a longer loop1 than other PL7 subfamily members (Figure 6B-i). In addition, our docking result suggested that this loop1 may engage in the catalysis of the substrate through stabilizing the substrate-lyase complex (His^376^ and Arg^380^). Further analysis revealed that certain amino acid residues may influence the catalysis by interacting with His^376^ and Arg^380^. For example, Ser^375^ can form a hydrogen bond with His^376^, and Arg^380^ shows interactions with Glu^377^ and Gln^495^ by hydrogen bonding (Figure 6B-ii).

### 3.5 Enzymatic kinetic analysis of VaAly2 and its mutants

**FIGURE 7:**
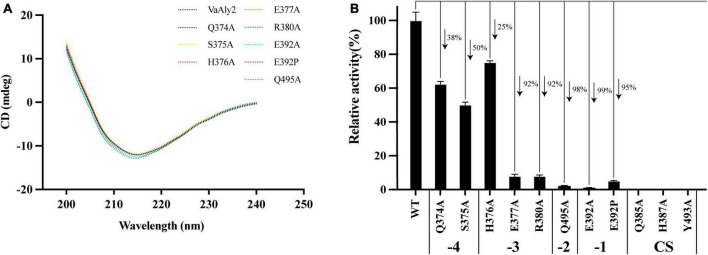
Effect of different residue mutations on the protein structure and alginolytic activity. **(A)** Circular dichroism spectra of WT and mutants. **(B)** Enzyme activities toward sodium alginate of different mutants.

According to the docking analysis and structure alignment, the structurally guided alanine screening mutations of VaAly2 (except for E392P) were constructed to understand their roles in catalytic efficiency. To ensure no secondary structure changes, mutants and WT were first detected by circular dichroism spectra. As shown in Figure 7A, difference between the WT and mutants was hardly observed, indicating that there was no difference in the main chain structures of the mutants. However, alanine substitution of amino acid residues in the active center significantly reduced the specific activity toward sodium alginate (Figure 7B). Consistent with other alginate lyases in the PL7 family, the residues (Gln^385^, His^387^, Tyr^493^) in the active center site are critical for alginolytic activity, and no activity was shown when they were mutated to alanine, respectively. The specific residue Glu^392^ at the −1 site limited in the PL7_5 subfamily contributed greatly to the high catalytic activity of VaAly2. When it was changed to alanine, little activity was observed. Since in the whole PL7 family, the common residue corresponding to Glu^392^ is proline, we wondered whether proline was able to recover the VaAly2 activity. As shown in Figure 7B, mutant E392P only reached 5% of the activity of the WT, indicating that Glu^392^ might act as an important marker for the PL7_5 subfamily. In addition, the newly identified residues (His^376^ and Arg^380^) predicted to be involved in alginate degradation were also mutated to alanine. The activity of the mutant H376A at the −3 site was decreased by 25% compared to that of the WT, while the activity of the mutant R380A was significantly reduced. To further understand the role of His^376^ and Arg^380^, the variants likely to hydrogen bond with His^376^ and Arg^380^ were also constructed and investigated. When the amino acid residue Ser^375^ interacting with His^376^ was mutated, approximately 50% reduction in the activity was observed (Figure 7B). Mutation of Glu^377^ or Gln^495^ interacting with Arg^380^ resulted in a more than 90% decrease of the activity, suggesting that Glu^377^ and Gln^495^ are needed for the high activity of VaAly2. In addition, the residue Gln^374^ in the loop1 also formed a hydrogen bond with Glu^377^, and its mutation led to a 38% reduction of the alginolytic activity, again indicating that Glu^377^ is essential for the high activity of VaAly2. Taken together, our data suggested that Arg^380^ in the long loop1 is required for the enzyme activity and it could interact with Glu^377^ and Gln^495^ by hydrogen bonding, thereby maintaining a stable binding of the substrate at subsites −3 and −2.

To further elucidate the mechanism of amino acid mutants affecting enzymatic activity, enzymatic parameters of the WT and mutants toward sodium alginate were quantified (Table 2). The *K*_*m*_ value of the mutant E392A was much higher than that of the WT, suggesting Glu^392^ is likely involved in the binding of sodium alginate. When alanine was changed to proline, the affinity was slightly increased. The ratio of *k*_cat_ to *K*_*m*_ of WT was higher than that of the mutants E392A and E392P, and the value of E392P was 150% higher than that of E392A, indicating that Glu^392^ is critical to a high catalytic efficiency, which can be partially complemented by proline. The *K*_*m*_ values of Q495A, R380A, and E377A were 180, 106, and 105% higher than that of the WT, respectively, suggesting their role in the binding of sodium alginate. The affinity of H376A was slightly decreased. The reduced catalytic efficiency of the mutants Q374A and S375A was mainly caused by lower affinity for sodium alginate, and decreased by 57% and 64%, respectively.

**TABLE 2:** Kinetic parameters of VaAly2 and its mutants toward sodium alginate.

Subsite	Enzyme	*K*_*m*_ (mg/mL)	*V*_max_ (nmol⋅s^–1^*CPSTABLEENTER*⋅mg^–1^)	*k*_cat_ (s^–1^)	*k*_cat_/*K*_*m*_ (s^–1^mg ^–1^ mL)
	VaAly2	0.5333 ± 0.0958	830.9 ± 37.2	47.88 ± 2.14	89.78
−4	Q374A	0.8290 ± 0.0243	550.8 ± 39.9	31.74 ± 2.30	38.29
−4	S375A	1.238 ± 0.138	694.4 ± 24.5	40.02 ± 1.41	32.32
−3	H376A	1.056 ± 0.156	786.8 ± 36.2	45.34 ± 2.08	42.93
−3	E377A	1.091 ± 0.268	344.4 ± 31.2	19.84 ± 1.80	18.18
−3	R380A	1.098 ± 0.141	248.2 ± 9.38	14.30 ± 0.54	13.02
+ 1	E392A	1.660 ± 0.398	141.0 ± 11.7	8.12 ± 0.67	4.89
+1	E392P	1.266 ± 0.178	269.8 ± 12.4	15.54 ± 0.71	12.27
−2	Q495A	1.494 ± 0.358	214.1 ± 17.5	12.33 ± 1.00	8.25

## 4 Discussion

VaAly2 from *V. alginolyticus* ATCC 17749 harbors two major domains, one N-terminal CBM32 domain, and one catalytic domain belonging to the PL7 family, which are linked by a helix. In addition, a signal peptide is present which is likely used for the location of VaAly2. In the PL7 alginate lyase Aly01 from *Vibrio natriegens* SK42.001, the signal peptide can be recognized by *E. coli* and thus, the protein was secreted to culture. When the signal peptide was removed from Aly01, the protein was only expressed in the cytoplasm (Meng et al., 2021). Similarly, the signal peptide in FlAlyA from *Flavobacterium* sp. strain UMI-01 also functions in *E. coli* and recombinant FlAlyA was observed in the periplasm (Inoue et al., 2014). The function of CBM is more versatile, such as thermostability, enzymatic activity, substrate binding and product distribution among different alginate lyases (Dong et al., 2014; Li et al., 2015; Sim et al., 2017; Lyu et al., 2018; Tang et al., 2022a,b; Ji et al., 2023). In addition, the linker between CBM and CD domain is also suggested to play an important role in alginolytic activity (Meng et al., 2021). In the future, the detailed mechanism of the linker is required to fully understand its role in the enzymatic activity.

Similar to other PL7 enzymes, the catalytic domain of VaAly2 is predicted to be folded as a β-jelly roll (Xu et al., 2018) and contains a Gln^385^-His^387^-Tyr^493^ functional motif essential for alginate digestion (Kim et al., 2011; Zhu and Yin, 2015; Zhang et al., 2021). Compared to other reported alginate lyases (Cheng et al., 2020), VaAly2 from *V. alginolyticus* ATCC 17749 displays comparatively high activities toward different alginate substrates and acts as a bifunctional lyase due to its capability of catalyzing polyM and polyG. Salt is required for its alginolytic activity, which is widely observed in different alginate lyases. For example, in Aly1281 from *Pseudoalteromonas carrageenovora* ASY5, Na^+^ had positive effect on the substrate binding and alginolytic activity (Zhang et al., 2020). In the newly identified PL7_6 alginate lyase AlyC3 from *Psychromonas* sp. C-3, Na^+^ plays an important role in the aggregation of AlyC3, the sole PL7 alginate lyase functioning as a dimer (Xu et al., 2020). The crystal structure of AlyA1_*PL*7_ from *Z. galactanivorans* contains two calcium ions, which was proposed to bind negatively charged amino acid residues to an acidic substrate (Thomas et al., 2013). However, the role of different metals in the alginolytic activity is still largely unknown.

Further phylogenetic analysis revealed that VaAly2 is classified as a member of the PL7_5 subfamily. Compared to other PL7 subfamily members, some conserved amino acids at the center site (e.g., Glu^392^ in VaAly2) in PL7_5 enzymes are varied. Site-directed mutagenesis demonstrated that Glu^392^ is essential to the high alginolytic activity of VaAly2. Even if it was changed to proline, the common amino acid residue in the PL7 family, the activity of VaAly2 toward sodium alginate was significantly reduced, suggesting a critical role of Glu^392^ in alginate decomposition. Since it is completely conserved in the PL7_5 subfamily, Glu^392^ is proposed to be one hallmark for this subfamily. In addition, one loop (loop1) around the active site displays different sizes and shapes among different PL7 subfamily members, and PL7_5 enzymes contain a longer loop1. Although two amino acid residues (His^376^ and Arg^380^) were proposed to be involved in alginate digestion, Arg^380^ appears to be more important because its mutation resulted in a large loss of the alginolytic activity. Moreover, when amino acid residues predicted to interact with Arg^380^ were mutated, the activity of VaAly2 was also dramatically decreased. Therefore, the long loop1 is closely related to the high alginolytic activity of VaAly2, and the residues His^376^ and Arg^380^ are required for the interaction with the substate. Compared to His^376^, the key amino acid residue Arg^380^ is more critical, which together with the residues Glu^377^ and Gln^495^ might be involved in the maintenance of a stable binding of the sugar at the sites of −3 and −2. The mutation of either of them could lead to a nearly complete loss of the enzyme activity. On the other hand, loop1 participates in the product distributions, and enzymes with a longer loop1 produce smaller alginate oligomers (Dp < 4) (Zhang et al., 2022). In line with this, the major product of VaAly2 with a longer loop1 is Dp3.

In conclusion, our work provides new insight into alginate lyases belonging to the PL7_5 subfamily. Some specific characteristics of VaAly2 have been revealed, such as conserved residues at the active center limited in PL7_5 and critical loop1 for alginolytic activity. However, it remains unclear how these characteristics affect the catalytic efficiency in detail. A comprehensive analysis of the specific characteristics of different PL7 subfamilies will facilitate studies to distinguish multiple PL7 alginate lyase members.

## Data availability statement

The datasets presented in this study can be found in online repositories. The names of the repository/repositories and accession number(s) can be found below: https://www.ncbi.nlm.nih.gov/genbank/, AGV20271.1; https://www.uniprot.org/, A0A2I3CRV5.

## Author contributions

MD: Conceptualization, Data curation, Formal analysis, Investigation, Methodology, Software, Writing—original draft, Writing—review and editing. XL: Methodology, Supervision, Validation, Writing—review and editing. WQ: Funding acquisition, Writing—review and editing. YL: Conceptualization, Data curation, Formal analysis, Investigation, Supervision, Writing—original draft, Writing—review and editing. LW: Supervision, Writing—review and editing.

## Footnotes

1
https://smart.embl.de

2
https://services.healthtech.dtu.dk/services/SignalP-6.0/

3
https://www.expasy.org

4
http://www.clustal.org/clustal2/

5 http://espript.ibcp.fr/ESPript/cgi-bin/ESPript.cgi (accessed May 29, 2023).

6
http://www.cazy.org/

7
http://swissmodel.expasy.org

8
https://github.com/deepmind/alphafold

9
http://cloud.sdu.edu.cn/portal/view/main.html

10
http://weblogo.berkeley.edu/logo.cgi
