# Supplementary material for: Identification and characterization of a critical loop for the high activity of alginate lyase VaAly2 from the PL7_5 subfamily
Source: Front Microbiol. 2024 Jan 12;14:1333597. doi: 10.3389/fmicb.2023.1333597 (PMC10811132; doi:10.3389/fmicb.2023.1333597)
Supplement: Supplementary file 1 [file Data_Sheet_1.docx]

***Supplementary Material***

Identification and characterization of a critical loop for the high activity of alginate lyase VaAly2 from the PL7_5 subfamily

**Muxuan Du^1, 2†^, Xue Li^1†^, Weipeng Qi^3^, Yingjie Li^1*^, Lushan Wang^1^**

^1^State Key Laboratory of Microbial Technology, Shandong University, Qingdao, China

^2^School of Life Sciences, Shandong University, Qingdao, China

^3^Foshan Haitian (Gaoming) Flavoring& Food Co. Ltd., Foshan 528500, China

Table S1 Strains and plasmids used in this study

| **Strain or plasmid** | **Description** | **Source or reference** |
| --- | --- | --- |
| **Strains** |  |  |
| *Vibrio alginolyticus* ATCC 17749 | Wild-type | (1) |
| *E. coli* DH5α | The host strain used for general cloning | Dingguo (China) |
| *E. coli* BL21(DE3) | The host strain used for recombinant protein production | Tsingke (China) |
| **Plasmids** |  |  |
| pET-28a (+) | Km^r^, vector for the expression of proteins in *E. coli* | Novagen (Darmstadt, Germany) |
| pDMX01 | pET-28a (+) plus *VaAly2* | This study |
| pDMX02 | pET-28a (+) plus *VaAly2^Q374A^* | This study |
| pDMX03 | pET-28a (+) plus *VaAly2^S375A^* | This study |
| pDMX04 | pET-28a (+) plus *VaAly2^H376A^* | This study |
| pDMX05 | pET-28a (+) plus *VaAly2^E377A^* | This study |
| pDMX06 | pET-28a (+) plus *VaAly2^R380A^* | This study |
| pDMX07 | pET-28a (+) plus *VaAly2^Q385A^* | This study |
| pDMX08 | pET-28a (+) plus *VaAly2^H387A^* | This study |
| pDMX09 | pET-28a (+) plus *VaAly2^E392A^* | This study |
| pDMX10 | pET-28a (+) plus *VaAly2^E392P^* | This study |
| pDMX11 | pET-28a (+) plus *VaAly2^Y493A^* | This study |
| pDMX12 | pET-28a (+) plus *VaAly2^Q495^* | This study |

Table S2 Primers used in this study

| **Primers** | **Sequence (5'-3')** |
| --- | --- |
| **Construction of Expression Plasmid** | |
| *VaAly2*-F | CCGGAATTCTGTAATAGTACGGCGACCAT |
| *VaAly2*-R | CCGCTCGAGCTAGCCTTGGTACTTACCAA |
| **Generation of site-directed mutagenesis (Site directed mutagenesis bases were underlined)** | |
| *VaAly2^Q374A^*-F | AACTGGTGCGTCGCATGAAGTTGGCCGCTTTATTATCGGC |
| *VaAly2^Q374A^*-R | CTTCATGCGACGCACCAGTTGTGGTTGCGTGGTCAATCTT |
| *VaAly2^S375A^*-F | TGGTCAGGCGCATGAAGTTGGCCGCTTTATTATCGGCCAG |
| *VaAly2 ^S375A^*-R | CAACTTCATGCGCCTGACCAGTTGTGGTTGCGTGGTCAAT |
| *VaAly2^H376A^*-F | TCAGTCGGCTGAAGTTGGCCGCTTTATTATCGGCCAGATT |
| *VaAly2^H376A^*-R | GGCCAACTTCAGCCGACTGACCAGTTGTGGTTGCGTGGTC |
| *VaAly2^E377A^*-F | GTCGCATGCAGTTGGCCGCTTTATTATCGGCCAGATTCAT |
| *VaAly2^E377A^*-R | AGCGGCCAACTGCATGCGACTGACCAGTTGTGGTTGCGTG |
| *VaAly2^R380A^*-F | AGTTGGCGCCTTTATTATCGGCCAGATTCATGACAAAGAT |
| *VaAly2^R380A^*-R | CGATAATAAAGGCGCCAACTTCATGCGACTGACCAGTTGT |
| *VaAly2^Q385A^*-F | TATCGGCGCGATTCATGACAAAGATGATGAGCCAATTCGT |
| *VaAly2^Q385A^*-R | TGTCATGAATCGCGCCGATAATAAAGCGGCCAACTTCATG |
| *VaAly2^H387A^*-F | CCAGATTGCTGACAAAGATGATGAGCCAATTCGTCTTTAT |
| *VaAly2^H387A^*-R | CATCTTTGTCAGCAATCTGGCCGATAATAAAGCGGCCAAC |
| *VaAly2^E392A^*-F | AGATGATGCGCCAATTCGTCTTTATTATCGTAAGCTACCA |
| *VaAly2^E392A^*-R | GACGAATTGGCGCATCATCTTTGTCATGAATCTGGCCGAT |
| *VaAly2^E392P^*-F | AGATGATCCGCCAATTCGTCTTTATTATCGTAAGCTACCA |
| *VaAly2^E392P^*-R | GACGAATTGGCGGATCATCTTTGTCATGAATCTGGCCGAT |
| *VaAly2^Y493A^*-F | CGGTGTCGCTAACCAGAATATGTACGGTAATCCAGATGAT |
| *VaAly2^Y493A^*-R | TATTCTGGTTAGCGACACCGGCTTTAAAGTACATATATCT |
| *VaAly2^Q495A^*-F  *VaAly2^Q495A^*-R | CTATAACGCGAATATGTACGGTAATCCAGATGATTACGCA  CGTACATATTCGCGTTATAGACACCGGCTTTAAAGTACAT |


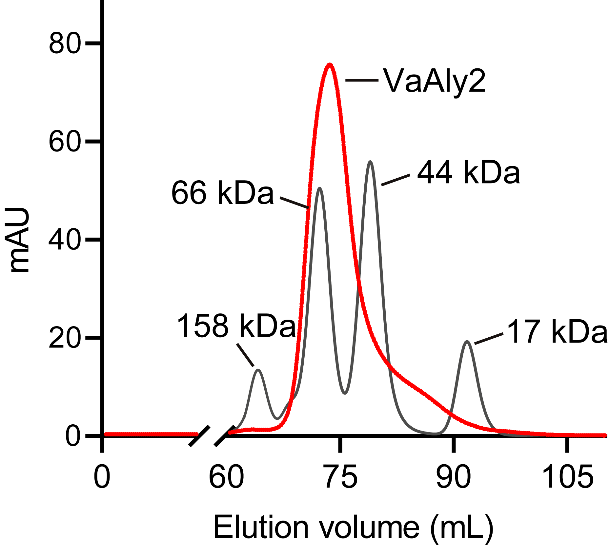


**Figure S1** Size-exclusion analysis of the oligomeric state of VaAly2 in solution using myoglobulin (17 kDa), ovalbumin (44 kDa), human albumin (66 kDa), and IgG (158 kDa) as protein size standards.

**References**

Liu XF, Cao Y, Zhang HL, Chen YJ, Hu CJ. 2015. Complete Genome Sequence of *Vibrio alginolyticus* ATCC 17749T. Genome Announc 3: e01500-14.
